# Novel Approach for Obtaining Variable Domain of New Antigen Receptor with Different Physicochemical Properties from Japanese Topeshark (*Hemitriakis japanica*)

**DOI:** 10.3390/md21110550

**Published:** 2023-10-24

**Authors:** Tomofumi Nakada-Masuta, Hiroyuki Takeda, Kazuhisa Uchida

**Affiliations:** 1 Graduate School of Science, Technology and Innovation, Kobe University, 7-1-49 Minatojimaminamimachi Chuo-ku, Kobe 650-0047, Japan; kazuhisa.uchida@port.kobe-u.ac.jp; 2 Bio-Diagnostic Reagent Technology Center, Sysmex Corporation, 4-3-2 Nishi-ku Takatsukadai, Kobe 651-2271, Japan; 3 Division of Proteo-Drug-Discovery Sciences, Ehime University Proteo-Science Center, Bunkyocho 3, Matsuyama 790-8577, Japan; takeda.hiroyuki.mk@ehime-u.ac.jp

**Keywords:** Japanese topeshark, VNAR, phage display, NGS, selection pressures

## Abstract

Diverse candidate antibodies are needed to successfully identify therapeutic and diagnostic applications. The variable domain of IgNAR (VNAR), a shark single-domain antibody, has attracted attention owing to its favorable physicochemical properties. The phage display method used to screen for optimal VNARs loses sequence diversity because of the bias caused by the differential ease of protein expression in *Escherichia coli*. Here, we investigated a VNAR selection method that combined panning with various selection pressures and next-generation sequencing (NGS) analyses to obtain additional candidates. Drawing inspiration from the physiological conditions of sharks and the physicochemical properties of VNARs, we examined the effects of NaCl and urea concentrations, low temperature, and preheating at the binding step of panning. VNAR phage libraries generated from Japanese topeshark (*Hemitriakis japanica*) were enriched under these conditions. We then performed NGS analysis and attempted to select clones that were specifically enriched under each panning condition. The identified VNARs exhibited higher reactivity than those obtained by panning without selection pressure. Additionally, they possess physicochemical properties that reflect their respective selection pressures. These results can greatly enhance our understanding of VNAR properties and offer guidance for the screening of high-quality VNAR clones that are present at low frequencies.

## 1. Introduction

Selecting antibodies that exhibit suitable physicochemical properties is key to developing candidate antibodies for diagnostics and therapeutics. During the initial stages of development, preparing diverse candidate antibodies will help increase the probability of obtaining clones that meet target specifications.

Single-domain antibodies (sdAbs) are derived from heavy-chain antibodies (hcAbs) derived from camels and sharks. The major variable domain of the heavy chain antibody (VHH) is the variable domain of camelid hcAb. VNAR (Variable domain of new antigen receptor) is the variable domain of immunoglobulin new antigen receptor (IgNAR), a shark-derived hcAb. SdAbs are attracting attention owing to their various physicochemical properties, such as thermal stability, reversibility, ease of multimerization, high-level expression in *Escherichia coli*, and access to buried epitopes that are unobtainable with full-body antibodies [1,2,3,4,5]. SdAbs may have advantages over conventional antibodies in novel therapeutics and diagnostics [6].

VNAR exhibits several favorable properties compared to VHH. VNAR is the smallest antibody molecule in the animal kingdom and lacks conventional CDR2, which is present in IgG and VHH [4]. Instead of lacking CDR2, VNAR has a longer CDR3 than other antibodies and additional diverse regions such as hypervariable region 2 (HV2) and hypervariable region 4 (HV4) [4,7]. Sharks are evolutionarily distant from mammals, including camels, in the phylogenetic tree; therefore, sharks can recognize highly conserved proteins as antigens and have the potential to generate VNARs against them [4]. VNARs are classified into several types according to the number and position of the noncanonical cysteine residues [8,9].

Research on the construction of VNAR, including artificial synthesis and semi-synthetic libraries, has been reported [4,10]. In contrast, screening methods have not been sufficiently studied, and phage display has been adopted as the most common screening method for discovering VNARs. Candidates that specifically bind to antigens can be isolated from phage libraries prepared from shark spleens [4,10]. However, it is not easy to apply screening methods for antibodies from species other than sharks to obtain VNARs because the antibody characteristics are significantly different.

Enrichment of clones via phage display methods is highly affected by expression bias in *E. coli*, resulting in the abundance of a few antibodies and low frequency of most others [11], which is a challenge for obtaining diverse candidate clones. This showed that the number of VNARs in the phage pool after panning was much higher than that obtained by random clone picking [11]. To solve this problem, methods have been developed to reach deep into phage pools [12].

The common method to overcome the decrease in antibody diversity is based on increasing the diversity of the phage library and distinguishing the differences in the performance of clones [13]. As mentioned above, while the former has been studied in terms of VNAR, research on the latter is rare. It is important to focus on the latter approach to optimize the VNAR acquisition method. One method to distinguish the differences in the performance of clones is to alter the selection pressure. Selection pressure in the phage display method to generate antibodies with the desired physicochemical properties has been investigated [14,15]. Preheating was investigated as a method to improve the thermal stability of the single-chain variable fragment (scFv) [14]. Although thermal stability and reversibility are attractive properties of VNAR [1,2], selection pressures, such as preheating, to improve the physicochemical properties of VNARs have not been sufficiently investigated.

In addition to changing the selection pressure, next-generation sequencing (NGS) technology has been utilized. NGS technology has greatly improved the ability to analyze genetic information at a relatively low cost in a short period [16]. Gene sequences of sdAbs were determined using the Illumina MiSeq system, as well as Fab and scFv [12]. To identify the positive phages and evaluate the enrichment process, NGS analysis was performed to characterize the phage library before and after several rounds of panning.

The selection of diverse clones has been investigated by combining changes in selection pressure and NGS analysis [12]. To optimize the method of generating diverse clones, it is important to consider the physicochemical properties of each antibody. The application of these techniques to obtain shark antibodies has not been fully investigated.

Cartilaginous fish have significantly different plasma compositions and optimal temperatures owing to their different living environments compared with other immunized host animals. Shark blood contains 340–500 mM NaCl and 300–400 mM urea for osmotic adjustment to approximately 1000 mOsm/kg [17]. Furthermore, because these cartilaginous fish are poikilotherms, their body temperature is the same as that of seawater. In general, sharks, which are ectothermic animals, have body temperatures equivalent to those of the seawater environment in which they live, which is generally lower than those of warm-blooded animals.

Here, we investigated the effect of selection pressure on panning using VNAR display phage libraries produced from Japanese topeshark (*Hemitriakis japanica*) immunized with Venus fluorescent protein [18]. Japanese topeshark is widely distributed in the waters around Japan, Taiwan, China, and Korea [19]. It is docile and safe, making it suitable for handling laboratory. It has been demonstrated that VNARs can be produced in Japanese topeshark and related species. Analysis of IgNARs from the banded houndshark (*Triakis scyllium*), production of a semi-synthetic VNAR library, and physicochemical analysis of isolated VNARs have been reported [20,21,22]. Kim et al. immunized banded houndsharks with SARS-CoV-2 spike RBDs to produce antigen-specific VNARs [23]. We immunized Japanese topesharks with the Venus protein and reported an increase in the Venus-specific IgNAR antibody titer [24]. We constructed a VNAR display phage library from immunized sharks, enriched the antigen-specific VNARs under general panning conditions, and isolated several VNAR clones.

In this study, we aimed to distinguish VNARs with unique properties based on the physiological characteristics of sharks and physicochemical properties of VNARs from a large number of other clones under the condition of reduced diversity due to expression bias in *E. coli*. We applied an approach that combines selection pressure and NGS analysis to VNAR screening using phage display technology and verified its usefulness in solving this problem. We used the library produced in a previous study and enriched the VNARs under various selection pressures [24]. The selection pressures examined included conditions that mimicked the unique blood environment of sharks (high salt concentration, high urea concentration, and low temperature) and conditions that leveraged their distinctive physical properties of VNARs (preheating). An overview of the enriched VNAR library was analyzed via deep sequencing using NGS. Analysis of physicochemical properties is important to verify the usefulness of our approach for VNAR acquisition. Since the VNAR screening method has been rarely investigated, the selected antibodies have been insufficiently evaluated from the viewpoint of physicochemical properties. Therefore, to verify the usefulness of our method, we analyzed the interaction with the antigen (ELISA, Isothermal Titration Calorimetry (ITC) and Surface Plasmon Resonance (SPR)) and thermal stability (Differential Scanning Calorimetry (DSC)) of the obtained clones. Based on the data obtained in this study, we discussed a novel selection method for industrially useful shark antibodies.

## 2. Results

### 2.1. Identification of Various Kinds of VNAR under Different Panning Condition

**Figure 1:**
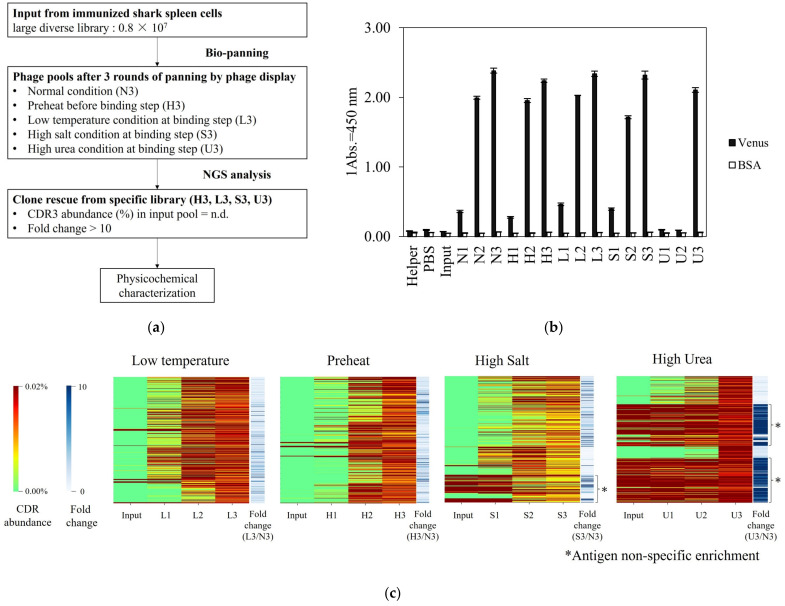
Identification of Venus-specific VNAR under different panning conditions. (**a**) An initial VNAR phage library (input) was used in multiple selection rounds against Venus and resulted in an enrichment of phage binding to the target molecule with five different selection pressures. Next-generation sequencing (NGS) enabled the sequence analysis of the input and the other pools after multiple panning. Based on CDR3 abundance and fold change of each pool, potential target-specific VNARs that exist at low abundance in N3 could be rescued and tested for a desired physicochemical property. (**b**) Phage ELISA of multiple panning against Venus (black columns) and BSA (white columns) was performed, and the absorbance of 450 nm for each pool was represented as mean. Error bars represent standard deviations (SDs) of triplicate measurement. (**c**) Heat map of CDR3 abundance (%) from input to round 3 of top 500 clones with the highest appearance frequency for each panning condition (red to green color). Fold change was colored from white to blue, next to CDR3 abundance.

As shown in Figure 1a, to obtain several VNARs that exhibited different physicochemical properties, we investigated five types of selection pressures: normal, preheating, low temperature, high salt concentration, and high urea concentration during the binding process of phage display. Three rounds of panning were performed under normal, preheated, low temperature, high salt concentration, and high urea concentration conditions. After panning, the library was named a phage pool according to the following rules: the panning condition is represented by the first letter, and the number of rounds is represented by the number of second letters. For example, the phage pool in round 3 under normal conditions was denoted as N3. Furthermore, the phage pool before panning was named “Input”. Phage ELISA was performed to evaluate antigen-specific enrichment. Phage particles specifically reacted with Venus but not with BSA (Figure 1b). The absorbance of the phage ELISA increased each round. Sufficient enrichment was observed after three rounds of panning under each condition.

For NGS analysis, phage pools from rounds 1 to 3 were prepared under five panning conditions. These 15 pools and inputs, totaling 16 pools, were subjected to NGS analysis. Sequences without stop codons were extracted from sequence reads >400 bp in length. CDR3 sequences were extracted from these sequences. The numbers of CDR3 reads are listed in Table S1. CDR3 abundance (%) is the quotient of the number of occurrences of each identified CDR3 sequence and the total number of CDR3 in each phage pool.

In round 3 pools, under each condition, the top 500 CDR3 sequences with high abundance were extracted and aligned. Subsequently, a heat map was generated to illustrate the prevalence of highly abundant CDR3 from input to round three, colored red to green, indicating low to high abundance (Figure 1c). The U3 pool had a large proportion of red in the input, suggesting that the VNARs present in the input were not sufficiently excluded until three rounds of panning. Some sequence groups in S3, marked with an asterisk in Figure 1c, were not eliminated from the input, although their proportion was smaller than that of U3. In contrast, the L3 and H3 pools eliminated most clones from the input pool during the panning process.

The fold-change was calculated to select preferentially enriched clones in each pool. The fold change was calculated as CDR3 abundance in each pool divided by CDR abundance in N3. Higher values indicate more condition-specifically enriched clones.

The fold changes in the top 500 sequences of each phage pool are shown in Figure 1c. Low to high fold changes are shown in white and blue, respectively. Clones with a fold change >10 in U3 were frequently observed. CDR3 abundance in these antibody groups was higher than 0.01% of the input pool. These sequencing groups were non-antigen-specifically enriched. In contrast, L3, H3, and S3 had relatively fewer sequence groups, as shown in blue, than U3. These sequence groups were not highly abundant in the input. Slight antigen-specific enrichment was detected under low temperature, preheating, and high salt concentration conditions, which was different from normal conditions. However, under high-salt conditions, a small number of non-antigen-specific concentrated VNARs, similar to the high urea concentration conditions, were included.

**Figure 2:**
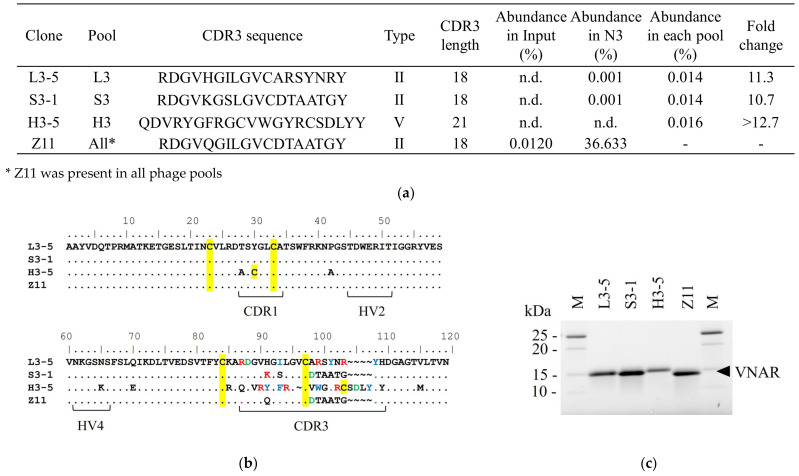
The feature of selected VNARs (**a**) The characterization of selected clone from each pool. n.d. = not detected. (**b**) VNAR amino acid sequence of selected clone. Complementarity determining region 1 (CDR1), Hyper variable region 2 (HV2), HV4 and CDR3 are expressed under the sequences. The sequences were expressed by using BioEdit version 7.2.5. Cysteine residues are highlighted in yellow. Positively charged amino acids, negatively charged amino acids, and hydrophobic amino acids in CDR3 are shown in red, green, and blue, respectively. (**c**) Sodium dodecyl-sulfate polyacrylamide gel electrophoresis (SDS-PAGE) of the selected clones under non-reducing condition. M: Protein Marker.

Figure 2a shows clones that satisfied the following criteria: CDR3 abundance in input = n.d., fold change > 10, and antigen-binding activity. No VNAR satisfying this criterion can be selected in the U3 pool. Although the enrichment patterns of L3, S3, and H3 were similar to those of N3, condition-specifically enriched clones were selected.

As shown in Figure 2a, the sequences of Z11 and the condition-specific clones were classified as type II, except for H3-5 [8]. CDR3 abundance in L3-5 was 0.014% in L3 and 0.01% in N3. Because the fold change was 11.3, L3-5 was specifically enriched at low temperatures.

L3-5 and Z11 sequences differed by seven residues in CDR3 (Figure 2b). One of the characteristic differences between Z11 and L3-5 was the net charge. Position 98 of Z11 was a negatively charged amino acid, Asp, and in L3-5, the residue at the same position was a neutrally charged amino acid, Ala. Z11 has a neutral amino acid Thr at position 99 and Gly at position 103, whereas L3-5 has a positively charged Arg at both positions. The frequencies of the aromatic amino acids also differed. At position 101, Z11 was Ala, whereas L3-5 was Tyr.

The CDR abundance in S3-1 was 0.014% in S3 and 0.001% in N3. A fold change of 11.7 suggested that it was specifically enriched under high-salt concentration conditions (Figure 2a). S3-1 and Z11 differed in two residues. Position 91 of Z11 is Gln, and S3-1 at the same position is the positively charged amino acid Lys. At position 93, Z11 is the hydrophobic amino acid Ile and S3-1 is the hydrophilic amino acid Ser.

CDR3 abundance in H3-5 was shown to be as low as 0.016% in the H3 pool and was not detected in N3 (Figure 2a). A fold change of >12.7 suggested that H3-5 was specifically enriched under preheating conditions. The sequence contained two Cys residues in CDR3 and was therefore classified as type V [9]. Other clones had different CDR3 sequences and lengths. The number of aromatic amino acids (Ile, Tyr, Phe, and Trp) contained in CDR3 was one in Z11 and six residues for H3-5.

For industrial applications, antibody homogeneity is important for the development of production processes. To assess molecular homogeneity, SDS-PAGE of the purified VNAR was performed (Figure 2c). A single band was observed at a molecular weight of approximately 15 kDa in all clones. The purity of VNARs was high. A subsequent evaluation of the physicochemical properties of the purified antibodies was also presented.

### 2.2. Comparison of the Reactivity to the Antigen under Each ELISA Condition

To assess the feasibility of acquiring candidate clones with different characteristics, we compared the reactivity of the condition-specifically enriched VNARs with that of Z11. ELISA was performed to investigate the behavior of each clone under specific conditions. After measuring the absorbance at 450 nm at several antibody concentrations, the fitting was performed with a 4-parameter logistic regression model using Softmax 6.5.1 to calculate EC_50_ [25].

**Figure 3:**
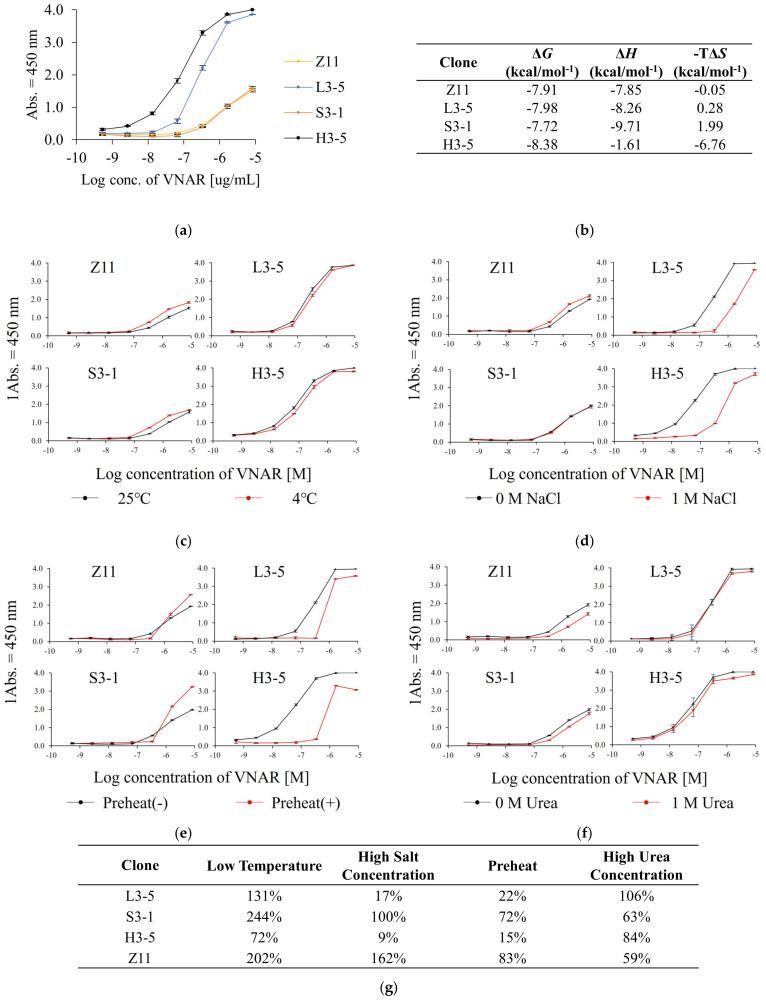
The reactivity analysis under each ELISA condition. (**a**) ELISA result under normal ELISA condition. (**b**) Isothermal titration calorimetry (ITC) data for binding of each VNAR titrated with Venus. The reactivity comparison between normal ELISA condition and (**c**) low temperature (4 °C), (**d**) high NaCl concentration, (**e**) preheat and (**f**) high urea concentration condition. Error bars represent standard deviations (SDs) of triplicate measurement. (**g**) The relative reactivity under each ELISA condition in comparison with that under normal ELISA condition.

We compared the reactivity of Z11 and the condition-specifically enriched VNARs by ELISA under normal conditions (Figure 3a). The EC_50_ under normal conditions was 3.0 × 10^−7^, 1.3 × 10^−6^, 9.0 × 10^−8^ and 1.3 × 10^−6^ M for L3-5, S3-1, H3-5 and Z11, respectively. The selected clones showed reactivity equal to or higher than that of Z11. Clones present at a low frequency in each pool showed higher antigen reactivity than those detected by the conventional method, demonstrating the practicality of this method. Figure 3b shows the ITC analysis data. Figure S1 shows the details of the ITC analysis. Z11, L3-5, and S3-1 were enthalpy-driven interactions, whereas H3-5 was entropy-driven.

Subsequently, the influence of selection pressure on the physicochemical properties of the selected VNAR was evaluated by calculating its relative reactivity. Relative reactivity was calculated as the quotient of the EC_50_ under normal ELISA conditions divided by the EC_50_ under each ELISA condition. A value exceeding 100% indicated an improvement in reactivity under each ELISA condition compared to that under normal ELISA conditions.

Figure 3c displays the effect of temperature change on the reactivity. When the reaction temperature changed from 25 °C to 4 °C, the relative reactivities were 131%, 244%, 72%, and 202% for L3-5, S3-1, H3-5 and Z11, respectively (Figure 3g). All the clones showed equal or higher reactivity under low-temperature conditions than under normal conditions. L3-5 and Z11 were confirmed to be enriched in the L3 pool, and their reactivity was slightly improved by ELISA at low temperatures, demonstrating that panning at low temperatures was reflected in the characteristics of the clones.

Next, we evaluated the effect of salt concentration on the reactivity (Figure 3d). When the NaCl concentration of the reaction solution was changed from 0 to 1 M, the relative reactivities were 17%, 100%, 9%, and 162% for L3-5, S3-1, H3-5, and Z11, respectively (Figure 3g). Z11 and S3-1 retained their reactivity even in 1 M NaCl, whereas L3-5 and H3-5 showed significantly decreased reactivity. S3-1 and Z11 were enriched in the S3 pool, indicating that the panning conditions were reflected in the physicochemical properties of the clones with high salt tolerance.

The results of the reactivity comparison with and without preheating are shown in Figure 3e. The relative reactivities of L3-5, S3-1, H3-5, and Z11 were calculated as 22%, 72%, 15%, and 83%, respectively (Figure 3g). Z11 and S3-1 did not substantially decrease antigen reactivity even after preheating. In contrast, the reactivity of L3-5 and H3-5 decreased significantly. Although H3-5 was isolated from the H3 pool, its relative reactivity after heating was remarkably reduced. Hence, the preheated panning condition was not necessarily reflected in antibody refolding after heat denaturation in ELISA under preheating conditions.

Finally, we investigated the effect of urea concentration on reactivity (Figure 3f). The relative reactivities of L3-5, S3-1, H3-5, and Z11 were 106%, 63%, 84%, and 59%, respectively (Figure 3g). No significant decrease in reactivity was observed for any of the VNARs.

### 2.3. Analysis of Thermal Stability and Reversibility

Thermal stability is related to aggregation risk and long-term storage stability [26,27]. Therefore, thermal stability is important for evaluating the developability of diagnostic reagents [28]. Thermal stability was evaluated by DSC to evaluate the effect of the selection pressure on the preheating conditions.

**Figure 4:**
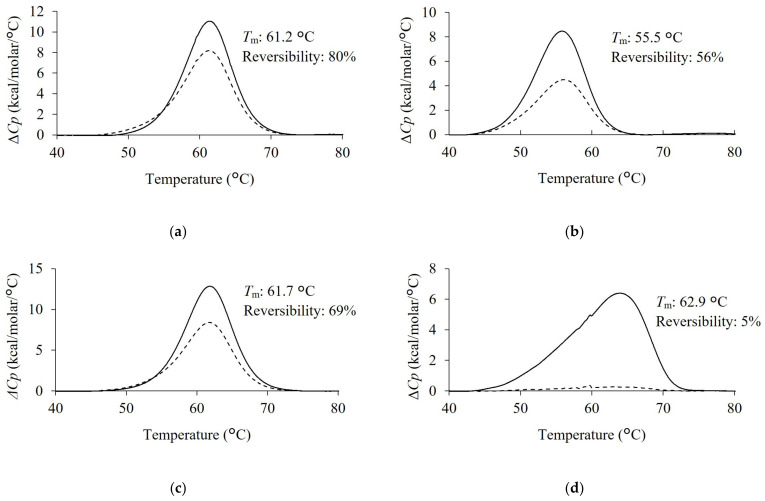
Thermogram of each VNAR clone. (**a**) Z11, (**b**) L3-5, (**c**) S3-1, (**d**) H3-5. The concentration of purified VNAR in PBS was set to 1 mg/mL. DSC measurement was performed at a scanning rate 1.0 °C/min from 30 °C to 90 °C. Solid lines indicate the first scan. Dotted lines indicate the second scan. *T*_m_ value and the reversibility were shown next to each peak.

Comparative data on the thermal stabilities of the condition-specifically enriched VNAR and Z11 were obtained (Figure 4). H3-5 had the highest *T*_m_ value among the antibodies tested in this study, showing 62.9 °C. Subsequently, the *T*_m_ of Z11 and S3-1 were 61.2 °C and 61.7 °C, respectively. *T*_m_ value of L3-5 was the lowest at 55.5 °C. *T*_m_ value of H3-5 was higher than that of Z11. Preheating treatment successfully improves the thermal stability of antibody molecules [14]. This result showed that the effect of pretreatment on phage display screening was reflected in an improvement in the *T*_m_ value of the screened VNARs.

The second scan of DSC measurement was performed to assess reversibility. The reversibility of Z11, L3-5, S3-1, and H3-5 were 80%, 56%, 69%, and 5%, respectively. H3-5 exhibited a slight endothermic peak in the second scan and the lowest reversibility among the selected clones. In contrast, Z11, L3-5, and S3-1 exhibited higher reversibility than H3-5. The reversibility values are as follows: Z11 > S3-1 > L3-5 > H3-5.

## 3. Discussion

In this study, additional antibody sequences were obtained using a combined approach of selection pressure and NGS analysis, which mimicked the unique physiological conditions of sharks and the physical properties of VNARs. The selected clones showed higher reactivity than abundant clones and adaptation to selection pressure.

We evaluated the effectiveness of these approaches in various VNAR candidates, even in the expression bias of *E. coli* in phage display selection. The abundance in each pool of the condition-specifically enriched clone ranged from 0.014% to 0.016% (Figure 2a). In contrast, the abundance of the condition-specifically enriched clone in N3 was much lower than that in each condition, suggesting that they were not enriched by panning under normal conditions (Figure 2a). These highly functional clones were relatively enriched and could be distinguished from a large number of other clones by panning under an appropriate selection pressure. The abundance of these condition-specifically enriched clones was too low to be selected by random pickup even in the pool of each panning condition, indicating the necessity of not only changing the selection pressure but also analysis using NGS. Taken together, we gained important insights into the guidelines for obtaining additional VNARs.

All selected VNARs showed resistance to low temperature and high urea concentration. Shark serum contains high concentrations of urea for osmotic control [17]. The body temperature of sharks in the living environment is 15–25 °C, which is lower than that of other immunized host animals. To find a panning strategy specific to the VNAR based on biomimetics, we examined panning conditions that mimicked the physiological conditions of sharks. S3-1 and H3-5, which were not enriched in the L3 pool, are resistant to low temperatures (Figure 3c). L3-5, S3-1 and H3-5, which were not selected by U3, retained antigen-binding affinity under high urea conditions (Figure 3f). These results suggested that resistance to low temperature and high concentration of urea are an inherited characteristic of VNAR. Affinity results of VNARs at low temperatures showed that the reactivity was improved mainly by improving *k*_off_ compared to 25 °C (Figure S2). In the case of VNAR, *k*_on_ increased slightly, and *k*_off_ increased remarkably with temperature. On the other hand, previous studies have shown that both *k*_on_ and *k*_off_ of molecules other than VNAR increase with increasing temperature [29,30,31]. Since the temperature range in which sharks live is below 25 °C, most of the VNARs present in serum are expected to exhibit higher binding activity at low temperatures. VNAR showed resistance not only to low temperature but also to 1 M urea (Figure 3f). In this study, 0.5 M urea exhibits protein denaturing effects [32]. It has also been reported that a high concentration of urea inhibits antigen–antibody reactions [33]. Sharks contain high concentrations of urea in their serum, and labile proteins are expected to denature [34]. VNARs are predicted to be inherently resistant to high urea concentrations. In the U3 pool, non-antigen-specific enrichment was observed (Figure 1c), and clones meeting the criteria could not be selected. If VNARs are inherently resistant to high urea concentration, the addition of excess urea during panning is not necessary for VNAR selection. Important findings regarding temperature and urea concentration were obtained in the setting of the panning conditions.

The salt concentration resistance differed among VNARs. Shark serum contains high concentrations of NaCl for osmotic control [17]. From the results in Figure 3d, S3-1 and Z11, which could be selected from the S3 pool, retained their reactivity even in 1 M NaCl. CDR3 of S3-1 and Z11 contained less charged amino acids compared to the other two VNAR clones, suggesting that S3-1 and Z11 were less affected by electrostatic shielding [35,36]. Hence, the low usage of charged amino acids in CDR3 is important for high salt concentration resistance (Figure 2a,b and Figure 3d). On the other hand, the results of the phage enrichment process under high NaCl concentration conditions indicated that a small number of non-antigen-specific VNARs was observed under high salt concentration conditions (Figure 1c). Therefore, it is important to consider the appropriate salt concentration in order to obtain VNAR with high salt concentration resistance.

We investigated the effects of preheating conditions on the physicochemical properties of VNAR. The improved thermal stability of H3-5 is consistent with the results of a previous study, in which the thermal stability of target proteins was improved by preheating for phage display [14]. In terms of reversibility, the results for H3-5 are consistent with the irreversible aggregation of scFvs obtained via the phage display with preheating [37]. L3-5 and H3-5 contained more aromatic amino acids than Z11 (Figure 2b). As these amino acids have bulky side chains and hydrophobic rings, they contribute to aggregation by imparting rigidity to their structure [38]. It has been suggested that an abundance of aromatic amino acids is involved in the reduced reversibility upon preheating. This indicated that the selection pressure of preheating was reflected in the improvement of the *T*_m_ value of H3-5, whereas this selection pressure did not necessarily affect reversibility.

The reactivity of rare clones helps demonstrate the usefulness of this method for obtaining additional candidate clones. We considered the factors that contributed to the interaction between each clone and antigen. The condition-specifically enriched clones showed higher reactivity than Z11 under normal ELISA conditions, especially in L3-5 and H3-5. In ELISA, under high NaCl concentration conditions, Z11 showed no change in reactivity compared to salt-free conditions, whereas L3-5 and H3-5 showed a marked decrease in reactivity. As we mentioned above, this suggests that charged amino acids involved in these interactions are affected by electrostatic shielding [35,36]. In other words, charged amino acids were one of the factors responsible for the high reactivity of L3-5 and H3-5. The aromatic amino acids L3-5 and H3-5 were also used more frequently than Z11. H3-5 had an entropy change ΔΔ*H* of −6.71 kcal/mol compared to Z11 in ITC analysis, suggesting that H3-5 contributes to the interaction of hydrophobic amino acids. This is related to the fact that aromatic amino acids greatly contribute to interactions and affinities [39]. In S3-1, which had almost no difference in reactivity compared to Z11, the change in H91 from Z11 to Lys resulted in enthalpy gain, and the change of hydrophobic amino acid I93 to Ser caused entropy loss, resulting in enthalpy–entropy compensation, and no significant difference in antigen reactivity was observed [40].

It is essential to prepare antibodies with different properties for the development of diagnostics and therapeutics. The unique properties of the antibodies obtained in this study may possibly be utilized in next-generation applications. Some diagnostic marker antigens are bound by neutralizing antibodies in the human serum to competitively inhibit diagnostic raw materials. Pretreatment was required to remove inhibitory substances present in the sample [41]. Therefore, VNARs that can bind to antigens even at high concentrations of NaCl and urea, where the reactivity of conventional antibodies is significantly reduced, would promote the development of diagnostic agents without pretreatment.

## 4. Materials and Methods

### 4.1. Phage Display and Panning Method

The preparation of the anti-Venus VNAR phage library and purified Venus antigen has been described in a previous report [24]. The input phage library from immunized shark spleen cells was amplified. Input phages (1 × 10^13^) were added to an immune tube (Nunc, Wiesbaden, Germany) and blocked with skim milk to adsorb nonspecific binders. For only the preheat condition, the supernatant was heated at 70 °C for 30 min. After absorption, the supernatant was diluted 2-fold with phosphate-buffered saline (PBS), 2 M NaCl, or 2 M urea. Then, the supernatant was transferred to the Venus-coated well of the Maxisorp plate (Nunc, Wiesbaden, Germany) at 25 °C or 4 °C for 1 h. The plate was washed with PBS/0.05% (*v*/*v*) Tween 20 to remove the unbound phage particles. Venus-bound phages were eluted with Glycine-HCl pH2.2 and neutralized with 1/10 the volume of Tris-HCl pH 9.0. The eluted phage was then added to exponentially growing *E.coli* TG1 cells (OD 600 = 0.5). After incubating the *E. coli* cell mixture at 37 °C for 30 min, the cells were plated onto 2 × YT agar medium containing 1% (*w*/*v*) glucose and 100 µg/mL ampicillin, and then incubated at 30 °C overnight. The cells on the plate were collected, and the M13KO7 helper phage was used to induce phage production for the next round of selection.

### 4.2. Phage ELISA

Phage ELISA was conducted to evaluate the enrichment of antigen-specific phage particles. For this, 100 ug of Venus antigen was immobilized at 25 °C for 1 h and then blocked with 1% (*w*/*v*) skimmed milk-PBS at 25 °C for 1 h. The phages of each round of panning were diluted into 2 × 10^10^ phages in 100 µL of 1% (*w*/*v*) skimmed milk-PBS and then incubated in the wells at 25 °C for 1 h. After washing each well, an anti-M13 mAb horseradish peroxidase (HRP) conjugate (Abcam, ab50370, CB2, Cambridge, UK) diluted 1–3000 in 5% skimmed milk-PBS was added and then incubated at 25 °C for 1 h. ELISA was developed using a TMB substrate (Thermo Fisher Scientific, 34028, Waltham, MA, USA) and read at 450 nm.

### 4.3. NGS Analysis of VNAR Phage Pool and Clone Selection

Phagemids were extracted from *E. coli* stock of each phage pool using the Nucleospin Plasmid EasyPure kit (Takara Bio, Kusatsu, Shiga, Japan) and used as PCR templates for NGS analysis. Adaptor PCR was performed using a KAPA HiFi HotStart ReadyMix PCR kit (KAPA Biosystems, Wilmington, MA, USA). Sequencing was performed on an Illumina MiSeq sequencer (Illumina, San Diego, CA, USA) using the MiSeq Reagent Nano Kit v.2 (Illumina, San Diego, CA, USA). Sequences without stop codons longer than 400 bp were extracted from the deep-sequencing results. Sequences encoding VNAR were selected, and their CDR3 sequences were extracted. The CDR3 abundance was calculated by determining the ratio of the number of occurrences of each identified CDR3 sequence to the total number of CDR3 sequences in each phage pool. To identify the clones that were preferentially enriched under each selection pressure, the fold-change was calculated by dividing the CDR3 abundance in each enriched pool by the abundance in N3. The top 500 highly abundant CDR3 sequences were aligned using MAFFT [42], and heat maps were created using the heatmap function in the Seaborn Python package. VNAR candidates meeting the following criteria were selected as preferentially enriched clones: CDR3 abundance in input = n.d., fold change > 10, and antigen-binding activity. CDR3 sequence comparison figures were created using the alignment function (Clustal W [43]) in BioEdit version 7.2.5 (Bioedit, Manchester, UK).

### 4.4. Expression and Purification of VNAR

Next, VNAR was inserted into the expi293 expression vector (Thermo Fisher Scientific, Waltham, MA, USA). The vector was transfected into Expi293 cells (Thermo Fisher Scientific, Waltham, MA, USA) and cultured at 37 °C for 5 days. The supernatant was centrifugated at 1000 g for 10 min and filtration. His tag resin was added to the supernatant and incubated at 4 °C overnight in a rotator. The resin was washed with PBS and eluted with 300 mM imidazole/PBS. Subsequently, the eluent was purified using size exclusion chromatography (SEC). Two CV of PBS were eluted at a flow rate of 0.8 min/mL, and SEC purification was performed using Superdex 200 Increase 10/300 GL (Cytiva, Marlborough, MA, USA).

### 4.5. Evaluation of Purity by SDS-PAGE

For non-reducing 10% stain-free SDS-PAGE gel (Nacalai Tesque, Nakagyo-ku, Kyoto, Japan), the sample buffer solution without reducing reagent (×6) (Nacalai Tesque, Nakagyo-ku, Kyoto, Japan) was added to VNAR sample solution and heated at 95 °C for 5 min. The gel was run in 1 x SDS-running buffer (Nacalai Tesque, 12981-74, Nakagyo-ku, Kyoto, Japan) at 200 V for 30 min. The bands were detected using a GelDoc system and a UV/stain-free sample tray (Bio-Rad, Hercules, CA, USA).

### 4.6. Evaluation of the Reactivity under Each Condition by ELISA

EC_50_ was determined using 0.1 ug of Venus-coated well, blocked with 1% (*w*/*v*) BSA/PBS. Next, serial dilutions of purified VNAR were prepared 7 times in 50 μL of 1% (*w*/*v*) BSA/PBS. The sample solution was added and incubated at 25 °C for 1 h. After washing with 1% (*w*/*v*) BSA/PBS, Goat anti-6-His tag HRP-conjugated antibody (Bethyl Laboratories, A190-113P, Montgomery, TX, USA) diluted 1:10,000 in 1% BSA/PBS was added and incubated at 25 °C for 1 h. TMB substrate (Thermo Fisher Scientific, Waltham, MA, USA) was added and incubated for 10 min. The optical density was measured at 450 nm after termination of the reaction through the addition of 100 μL of 1 M H_2_SO_4_. Fitting was performed using a 4-parameter logistic regression model with Softmax 6.5.1 to calculate EC_50_.

### 4.7. Isothermal Titration Calorimetry (ITC)

ITC was performed using a MicroCal PEAQ-ITC automated system (Spectris, London, UK). All measurements were performed on degassed samples immediately before use. To investigate the binding of Venus to VNAR, experiments were performed with 50 μM Venus in the cell and 500 μM VNAR in the syringe. VNAR injections were carried out over a 15 s period with a 4 min equilibration period between each injection. Data analysis was performed using the ITC data analysis software Microcal Origin v1.21 (Spectris, London, UK).

### 4.8. Differential Scanning Calorimetry (DSC)

DSC measurement was evaluated using Microcal PEAQ-DSC (Spectris, Aldwych, London, UK) at a scanning rate 1.0 °C/min from 30 °C to 90 °C. The concentration of VNAR in PBS was adjusted to 1 mg/mL. The VNAR thermogram was normalized by subtracting the response of the PBS buffer. The second scan was analyzed under the same conditions as the 1st scan after the temperature was lowered from 90 °C to 30 °C. The DSC data were analyzed using a non-two-state model to obtain values for each thermostability parameter. The analysis was performed using the MicroCal PEAQ-DSC software v1.53 (Spectris, Aldwych, London, UK).

### 4.9. Surface Plasmon Resonance (SPR)

The kinetics of binding and dissociation between VNAR and Venus were measured using Biacore 8 K (Cytiva, Marlborough, MA, USA). VNAR-mouse Fc (2 μg/mL in HBS-EP buffer: 0.01 M HEPES pH 7.4, 0.15 M NaCl, 3 mM EDRA, and 0.005% *v*/*v* Surfactant P20) was injected to the Anti-mouse Fc antibody immobilized chip which was prepared using Mouse antibody capture kit (Cytiva, Marlborough, MA, USA). A single-cycle kinetic experiment was performed using a blank and five serial double-fold dilutions of Venus as the analyte. The binding and dissociation times were set to 120 s and 300 s, respectively. Regeneration was performed by injecting glycine-HCl (pH 1.7) for 30 s. Kinetic parameters were calculated using Biacore Insight Evaluation Software 2.0.15.12933 (Cytiva, Marlborough, MA, USA). 

## 5. Conclusions

In this study, we propose a novel method for preparing a diverse set of VNAR antibodies. We succeeded in discovering highly functional VNARs among many other clones by screening based on the physiological characteristics of sharks and the physicochemical properties of VNARs. VNARs selected using our method showed favorable physicochemical properties in terms of thermal stability and high antigen-binding activity under the buffer conditions of each selection pressure. These results suggested the utility of our approach for obtaining additional VNARs. Our findings suggest important screening guidelines for the commercialization of shark antibodies as additional high-performance VNARs.

## Data Availability

The data presented in this study are available upon request from the corresponding author.

## Supplementary Materials

The following supporting information can be downloaded from: https://www.mdpi.com/article/10.3390/md21110550/s1, Table S1: Results of next-generation sequencing (NGS) analysis of phage pools; Figure S1: Details of ITC analysis; Figure S2: Effect of temperature on affinity.

## Abbreviations

The following abbreviations are used in this manuscript.

sdAb	Single-domain antibody
hcAb	Heavy-chain antibody
VHH	Variable domain of the heavy chain antibody
VNAR	Variable domain of new antigen receptor
CDR	Complementarity determining region
HV2	Hypervariable region 2
HV4	Hypervariable region 4
scFv	Single-chain variable fragment
NGS	Next generation sequencing
N3	The phage pool in Round 3 under normal condition
L3	The phage pool in Round 3 under low-temperature condition
H3	The phage pool in Round 3 under preheat condition
S3	The phage pool in Round 3 under high salt concentration condition
U3	The phage pool in Round 3 under high urea concentration condition
ITC	Isothermal titration calorimetry
DSC	Differential scanning calorimetry
SPR	Surface plasmon resonance
*k*_on_	Association rate constant
*k*_off_	Dissociation rate constant
*T*_m_	Melting Temperature
Δ*G*	Gibbs free energy change
Δ*H*	Enthalpy change
Δ*S*	Entropy change
Δ*Cp*	The change in heat capacity
