# Supplementary material for: Novel Approach for Obtaining Variable Domain of New Antigen Receptor with Different Physicochemical Properties from Japanese Topeshark (Hemitriakis japanica)
Source: Mar Drugs. 2023 Oct 24;21(11):550. doi: 10.3390/md21110550 (PMC10672104; doi:10.3390/md21110550)
Supplement: Supplementary file 1 [file marinedrugs-21-00550-s001.zip › marinedrugs-2667715-supplementary.pdf]

Table S1. The result of NGS analysis of phage pools.

Condition	R0	R1	R2	R3
Input	75,451 (49,852)	-	-	-
Normal	-	61,218 (44,081)	133,114 (117,760)	90,064 (79,695)
Low temp	-	75,688 (53,680)	51,788 (41,630)	76,228 (42,372)
High salt	-	44,690 (33,708)	99,971 (87,566)	82,013 (74,217)
Preheat	-	67,390 (44,188)	129,833 (113,817)	44,321 (37,851)
High Urea	-	29,461 (19,790)	29,231 (18,116)	74,929 (39,971)

Summary of total number of CDR3 sequences and number of unique CDR3 sequences from input library to round 3 under each panning condition. Total number of CDR3 sequences was shown. Numbers between brackets indicated the number of unique CDR3 reads

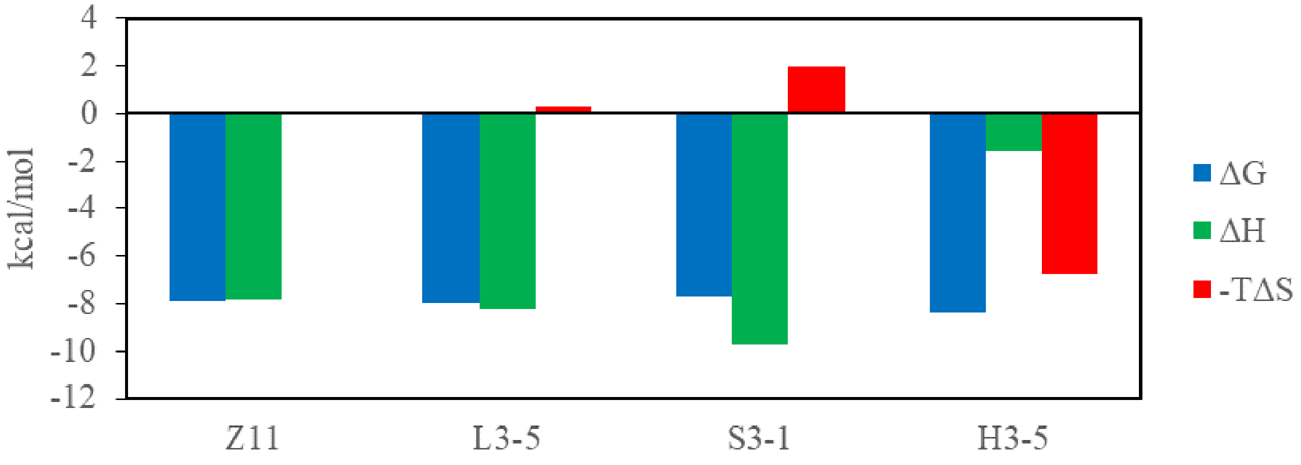

(a)

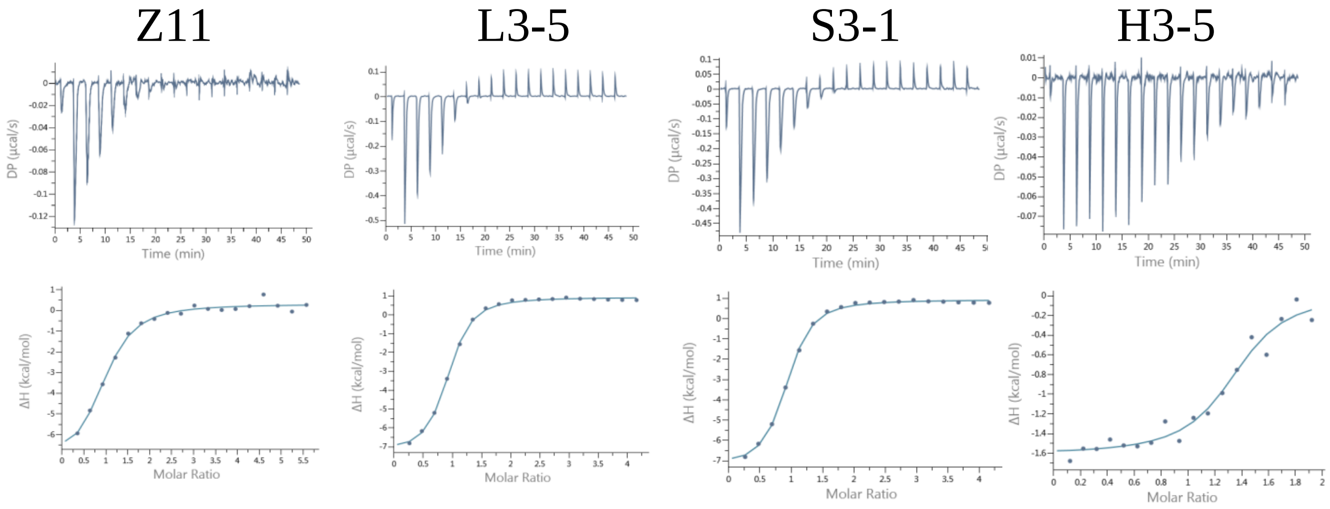

(b)

Figure. S1 The details of ITC analysis. (a) Thermodynamic signatures of four VNAR interactions. (b) ITC profile of the titration of Venus antigen with Z11, L3-5, S3-1, H3-5.

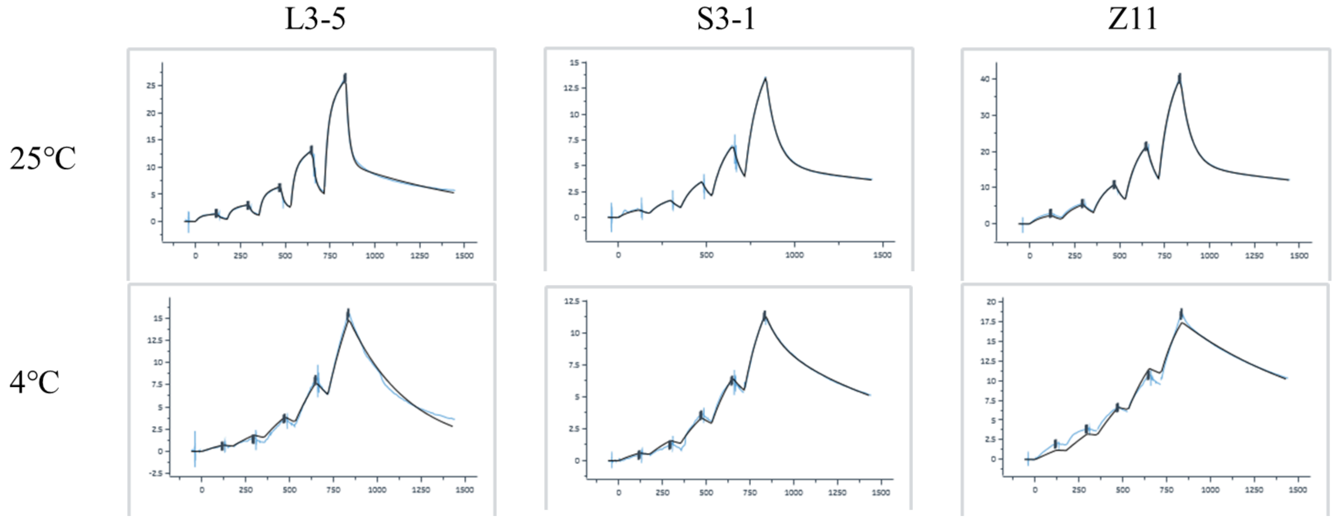

(a)

	25°C			4°C		
	k_{on} (1/Ms)	k_{off} (1/s)	K_D (M)	k_{on} (1/Ms)	k_{off} (1/s)	K_D (M)
L3-5	1.66×10^{3}	2.52×10^{-1}	1.51×10^{-4}	1.09×10^{3}	2.88×10^{-3}	2.64×10^{-6}
S3-1	1.13×10^{3}	1.44×10^{-2}	1.27×10^{-5}	2.64×10^{3}	6.25×10^{-3}	2.37×10^{-6}
Z11	2.74×10^{3}	1.50×10^{-2}	5.48×10^{-6}	1.35×10^{4}	3.02×10^{-3}	2.24×10^{-7}

(b)

Figure. S2 The effect of temperature on affinity. Kinetic parameters of L3-5, S3-1, and Z11 was shown. The kinetic parameter of H3-5 could not be determined because of aggregation during preparation (a) The SPR sensorgram. Curve fittings were shown by black line. (b) The analysis using single cycle kinetics was performed at 4 and 25°C
